# After the Visit: An Overview of Government and Community Programs Supporting Children with Medical Complexity

**DOI:** 10.3390/children4050035

**Published:** 2017-05-04

**Authors:** Kaitlyn B. Olson

**Affiliations:** Department of Pediatrics, Ann & Robert H. Lurie Children’s Hospital of Chicago, Northwestern University Feinberg School of Medicine, 225 E Chicago Ave, Box 152, Chicago, IL 60611, USA; kmolson@luriechildrens.org

**Keywords:** children with medical complexity, developmental disability, Early Intervention, legal rights, psychosocial support systems, special education, technology dependence

## Abstract

The optimal care of children with medical complexity (CMC) requires involvement from a network of professionals that includes physicians, nurses, ancillary service providers, and educators. Pediatric health care providers typically have early and frequent contact with the families of CMC. Therefore, they are in a unique position to connect families to developmental, educational, and psychosocial supports. This article reviews important government and community programs that support CMC living in the United States. It outlines the educational rights of children with disabilities and offers practical tips for collaborating with Early Intervention and the public school system. The article also provides an overview of financial assistance programs, respite care services, and support groups that are beneficial to CMC and their families.

## 1. Introduction

Children with medical complexity (CMC) are a heterogeneous group comprised of children with one or more chronic medical conditions which result in functional limitations, high health care utilization, and often the need for assistive medical technology [1]. For example, the classification of CMC would include both a child with hydrocephalus, global developmental delay, and a tracheostomy as well as a child with Trisomy 21, congenital heart disease, and gastrostomy tube dependence. With recent advances in pediatric medicine, the survival of CMC has increased [2]. Unfortunately, the families of CMC frequently report that they are unable to access the medical and ancillary services needed by their child [1].

Pediatric health care providers typically have early and frequent contact with the families of CMC. Therefore, they are uniquely positioned to connect families to developmental, educational, and psychosocial supports. Much attention has been paid to the patient-centered medical home as a tool to improve the medical care of CMC; however, there has been little guidance for physicians regarding the non-medical resources that benefit this population. This article reviews the current state of important government and community programs that support CMC living in the United States. It outlines the educational rights of children with disabilities and provides an overview of Early Intervention, the public school system, financial assistance programs, respite care services, and support groups that are beneficial to CMC and their families.

## 2. Early Intervention

The Individuals with Disabilities Education Act (IDEA) was passed by Congress in 1975 with the aim of improving the education of children with physical impairments and intellectual disabilities [3]. Part C of IDEA, passed in 1986, authorized the provision of developmental services to children with disabilities from birth until their third birthday [3]. This law led to the creation of the Early Intervention (EI) system.

EI programs are state-run and operate in all U.S. states as well as in U.S. territories. They provide developmental and medical supports including audiology, behavior therapy and psychological services, developmental therapy, occupational therapy, physical therapy, speech therapy, family training, social work, assistive medical technology, and service coordination [4]. Currently, more than 350,000 children receive services through EI [5]. Nearly all CMC aged 0–35 months will qualify to receive services through EI either based on an underlying medical condition or due to developmental delay.

Pediatric health care providers can and should refer CMC to EI or encourage the families to call for an intake evaluation. The contact person for EI referral varies by location; however, the Early Childhood Technical Assistance Center website (http://ectacenter.org/contact/ptccoord.asp) contains updated contact information to assist in this process [6]. Upon referral, the family will be contacted to obtain additional information and to consent for evaluation. If the EI program’s multidisciplinary evaluation determines that a child is eligible for services, an individualized family service plan (IFSP) will be created to outline the services that the child will receive. Services are typically provided in the home, but may be delivered in a daycare setting depending on the family situation. Many services (such as the child’s diagnostic evaluation and service coordination) are free; others may be subject to a sliding scale fee [3].

In addition to referring CMC to EI, pediatric health care practitioners can assist families by providing basic information about the EI evaluation process. With written permission from the family, physicians may facilitate the evaluation process by sharing pertinent medical information with the EI team [3]. If a family disagrees with their child’s IFSP, health care practitioners can encourage the family to communicate their concerns directly with the evaluating team—ideally before signing the IFSP. If the family remains dissatisfied with the IFSP, physicians can refer them to the local Protection and Advocacy Agency (P&A). P&As are federally funded organizations that provide advocacy and legal assistance to persons with disabilities. The Administration for Community Living website (https://acl.gov/programs/aidd/Programs/PA/Contacts.aspx) provides contact information for state P&As [7]. Health care practitioners can also refer families to the Center for Parent Information and Resources website (www.parentcenterhub.org, [8]) which contains helpful information about navigating the EI and public school systems as well as contact information for local Parent Training and Information Centers [3,8].

It is important for pediatric health care practitioners to realize that there are limitations to EI. Through EI, children are legally guaranteed to receive appropriate developmental services. However, due to a lack of funding and available resources, children may not receive the optimal frequency or intensity of these services. If the EI program is unable to provide what the health care practitioner or family feel to be a sufficient intensity of services, CMC may benefit from additional developmental support services obtained in the private sector. Pediatricians can help families by referring them to local organizations that provide these ancillary health care services.

## 3. Public School System

After a child’s third birthday, he/she is no longer eligible to participate in EI. At this time, the child must transition to the public school system for developmental and educational support services. Part B of IDEA and Section 504 of the Rehabilitation Act specify the educational rights of children with disabilities [3]. Both laws guarantee that all children, regardless of disability, are entitled to a free appropriate public education in the least restrictive environment [3]. Section 504 has a broader definition of disability than that used in IDEA and it applies to both public schools and private schools that accept federal funds [3].

Through the public school system, children with physical impairments and intellectual disabilities are eligible to receive educational support in the form of specialized instruction (in the general education classroom or in a special education classroom), therapy services (such as speech therapy and occupational therapy), medical services (such as nursing support for children with tracheostomies), physical accommodations (such as wheelchair ramps), and emotional health support (such as psychological services and behavior intervention planning). To receive services through the public school system, families of CMC should contact their child’s local public school to request an evaluation (if the child is enrolled in EI, the EI program will facilitate the transition process [3]). If a family is planning to enroll their child in a private school, the child is still eligible to receive a free evaluation from the public school system. The parents can use the resulting educational plan to determine where they believe that their child will receive the most educational support.

Upon completion of the public school system’s evaluation, a child may be deemed eligible for scholastic support through either an individualized education program (IEP) or a 504 plan. An IEP is a formal educational plan that outlines the scholastic support services that a child will receive, the educational setting in which the child will receive them, and the child’s specific learning goals. To be eligible for an IEP, a child must be classified as having one or more of the following: autism, hearing impairment, vision impairment, intellectual disability, traumatic brain injury, specific learning disability, speech or language impairment, emotional disturbance, orthopedic impairment, multiple disabilities, or other health impairment (that interferes with a student’s ability to learn) [3,9]. Most CMC will be eligible for an IEP. Children with disabilities who do not meet these criteria may be eligible for a 504 plan [9]. Similar to an IEP, a 504 plan is a legal document that outlines a child’s educational supports. In contrast, 504 plans are not subject to the same regulations that govern IEPs and there is significant variability in how the plans are created and structured.

If a child’s parents do not believe that the school district’s educational plan addresses their child’s needs, they may request modifications to the IEP or 504 plan. As with IFSP concerns, parents should be encouraged to voice their concerns directly to the child’s evaluation team. If a resolution cannot be reached, the child’s parents should contact their local P&A for assistance. Parents should be advised not to sign their child’s educational plan until their concerns have been addressed.

Pediatric health care practitioners can assist families by providing basic information about the school evaluation process. With written permission, they can share health information with the school to facilitate the creation of an appropriate educational plan [3]. When creating a medical plan for CMC, physicians should be aware that the public school system is only required to offer services that are sufficient to support the child’s educational needs [4]. Pediatricians should refer families to additional therapies if the services provided by the school are insufficient to promote the child’s overall health and well-being.

## 4. Financial Support Programs

The complex and intensive needs of CMC are costly both to the health care system and to families [10,11,12]. Fortunately, many of these families are eligible for financial support through government programs. Pediatric health care providers can assist families of CMC by providing them with basic information on the Supplemental Security Income (SSI) program and state Title V programs.

The SSI program, created through the Social Security Amendments of 1972, sends a monthly payment to families of children with disabilities [13]. To qualify, the child must have a physical and/or cognitive impairment that causes severe functional limitations [13]. The impairment must be anticipated to continue for at least one year or to result in the death of the child [13]. Additionally, families will only qualify for SSI if they meet specific income limitations [13].

SSI currently provides a payment of $735 per month for eligible individuals [14]. Depending upon the family’s state of residency, this payment may be supplemented by state funds [13]. In addition to a monthly payment, children in many states become automatically eligible for Medicaid if they receive SSI [13]. This provides additional medical insurance to families who would not have qualified for Medicaid due to their income level [13]. Families can apply for SSI by calling the Social Security Administration or visiting a Social Security office.

The Title V Maternal and Child Health Block Grant Program provides funding to states for programs addressing the health care needs of women and children [15]. Through Title V, states have established programs to improve the care of CMC; these programs include services such as care coordination and additional medical insurance. For example, Illinois has established the Division of Specialized Care for Children (DSCC) [16]. This program provides financial support as well as case management, care coordination, and transition planning services to CMC [16]. Vermont’s Children with Special Health Needs program provides financial assistance, care coordination, medical services, and respite care to CMC [17]. To learn more about a state’s Title V programs, visit the U.S. Department of Human Services Health Resources and Services Administration website [18].

## 5. Respite Care

Respite care provides either home-based or institutional services that allow families to take brief breaks from caring for a child with special health care needs [19]. Respite care has been shown to be highly valued by families of CMC [19]. Although these services can be difficult to access, the availability of respite care may be improving. In the last 10 years, Congress authorized the Lifespan Respite Care Program [20]. Through this program, 35 U.S. states have already received grants to support respite care [20].

Currently, families of CMC may be eligible to receive respite care services through 1915(c) Home and Community-Based Services waivers [21]. Given that these programs are established by individual U.S. states, the waiver programs vary in their eligibility criteria and services provided [21]. In addition, the ARCH National Respite Network and Resource Center has an online directory that includes links to state organizations and summer camps [22,23]. Some non-profit organizations, such as United Cerebral Palsy and Easterseals, also have local branches where trained providers offer respite care to CMC [23,24,25].

## 6. Support Groups

Family members of CMC experience high levels of stress and burnout [19]. Involvement in support groups has been consistently found to positively impact the mental and emotional health of caregivers [19,26,27,28]. Fortunately, it is now easier than ever for parents to locate both in-person and online support networks through the internet. Pediatric health care providers can support family members of CMC by encouraging them to connect with organizations related to their child’s medical challenges. For rare conditions, the National Organization for Rare Disorders website contains an extensive list of disease-specific organizations and support groups [29]. Physicians should also encourage parents to involve siblings of CMC in support groups, such as the Sibling Support Project [30].

## 7. Conclusions

Providing optimal care to CMC requires more than medical knowledge. Pediatric health care professionals can best support this population by familiarizing themselves with government and community programs that provide educational, financial, and psychosocial support services. Parents of CMC will be better prepared to advocate for their child with a basic understanding of his/her educational rights and the educational evaluation process. Their financial stress may be lessened through enrollment in government programs including the Supplemental Security Income program and state Title V programs. CMC and their families are also likely to receive psychosocial benefits from involvement in respite care and support groups.

Many of the support systems reviewed in this article are either federal programs or federal-state partnerships. The regulations governing the scope and funding of these programs change frequently. Therefore, while the article provides an overview of the current state of these services, it is important for pediatric health care providers to stay abreast of policy changes impacting these programs. By serving as a resource for basic information about these programs, physicians will have a greater impact on CMC and their families.
